# Retrospective Analysis of Training Intensity Distribution Based on Race Pace Versus Physiological Benchmarks in Highly Trained Sprint Kayakers

**DOI:** 10.1186/s40798-021-00382-y

**Published:** 2022-01-06

**Authors:** Manuel Matzka, Robert Leppich, Billy Sperlich, Christoph Zinner

**Affiliations:** 1 Integrative and Experimental Exercise Science and Training, University of Würzburg, Würzburg, Germany; 2 Software Engineering Group, Department of Computer Science, University of Würzburg, Würzburg, Germany; 3 Department of Sport, University of Applied Sciences for Police and Administration of Hesse, Wiesbaden, Germany

**Keywords:** Endurance training, Sprint kayaking, Training zones, Polarized training, Pyramidal training, Upper-body sport

## Abstract

**Background:**

Research results on the training intensity distribution (TID) in endurance athletes are equivocal. This non-uniformity appears to be partially founded in the different quantification methods that are implemented. So far, TID research has solely focused on sports involving the lower-body muscles as prime movers (e.g. running). Sprint kayaking imposes high demands on the upper-body endurance capacity of the athlete. As there are structural and physiological differences between upper- and lower-body musculature, TID in kayaking should be different to lower-body dominant sports. Therefore, we aimed to compare the training intensity distribution during an 8-wk macrocycle in a group of highly trained sprint kayakers employing three different methods of training intensity quantification.

**Methods:**

Heart rate (HR) and velocity during on-water training of nine highly trained German sprint kayakers were recorded during the final 8 weeks of a competition period leading to the national championships. The fractional analysis of TID was based on three zones (Z) derived from either HR (TID_Bla-HR_) or velocity (TID_Bla-V_) based on blood lactate (B_la_) concentrations (Z1 ≤ 2.5 mmol L^−1^ B_la_, Z2 = 2.5–4.0 mmol L^−1^ B_la_, Z3 ≥ 4.0 mmol L^−1^ B_la_) of an incremental test or the 1000-m race pace (TID_Race_): Z1 ≤ 85% of race pace, Z2 = 86–95% and Z3 ≥ 95%.

**Results:**

TID_Bla-V_ (Z1: 68%, Z2: 14%, Z3: 18%) differed from TID_Bla-HR_ (Z1: 91%, Z2: 6%, Z3: 3%) in each zone (all *p* < 0.01). TID_Race_ (Z1: 73%, Z2: 20%, Z3: 7%) differed to Z3 in TID_Bla-V_ (*p* < 0.01) and all three TID_Bla-HR_ zones (all *p* < 0.01). Individual analysis revealed ranges of Z1, Z2, Z3 fractions for TID_Bla-HR_ of 85–98%, 2–11% and 0.1–6%. For TID_Bla-V_, the individual ranges were 41–82% (Z1), 6–30% (Z2) and 8–30% (Z3) and for TID_Race_ 64–81% (Z1), 14–29% (Z2) and 4–10% (Z3).

**Conclusion:**

The results show that the method of training intensity quantification substantially affects the fraction of TID in well-trained sprint kayakers. TID_Race_ determination shows low interindividual variation compared to the physiologically based TID_Bla-HR_ and TID_Bla-V_. Depending on the aim of the analysis TID_Race_, TID_Bla-HR_ and TID_Bla-V_ have advantages as well as drawbacks and may be implemented in conjunction to maximize adaptation.

## Key Points

- TID_Bla-HR_ reveals a pyramidal TID for Z1, Z2 and Z3 (91 ± 4%, 6 ± 2%, 3 ± 2%), whereas TID_Bla-V_ showed a TID with a tendency towards a polarized TID (68 ± 14%, 14 ± 8% and 18 ± 8%).
- Based on the present data, a combination of both TID_Bla-HR_ and TID_Bla-V_ may yield value for TID quantification in kayak sprinting with TID_Bla-HR_ implemented to determine Z1 and Z2 and TID_Bla-V_ for Z3.
- The analysis of TID_Race_ showed a pyramidal TID pattern (Z1: 73 ± 5%, Z2: 20 ± 5%, Z3: 7 ± 2%) on the group level.
- The most noticeable difference between the TID determination methods is the comparably low interindividual variation in TID for TID_Race_.

## Introduction

Endurance athletes structure their training intensity usually based on different intensity zone models [1–4]. Most commonly, a three-zone model is implemented to distinguish the distribution of training between low, moderate and high intensities. The zones are generally determined based on physiological parameters (e.g. maximum oxygen uptake [VO_2max_], blood lactate [B_la_]) established from incremental testing. Zone (Z) 1 defines intensities below the aerobic threshold (< 2.5 mmol L^−1^ B_la_ or ≤ 80% of VO_2max_), Z2 relates to intensities between aerobic and anaerobic threshold (2.5–4.0 mmol L^−1^ B_la_ or 81–87% of VO_2max_) and Z3 equals intensities above the anaerobic threshold (> 4.0 mmol L^−1^ B_la_ or ≥ 88% of VO_2max_) [5]. Different internal (e.g. heart rate [HR], VO_2_, blood lactate) and/or external (e.g. velocity, power output) parameters are continuously monitored to quantify these intensity zones during daily training.

Besides the consensus in research regarding the physiologically based zone demarcation, different TIDs have been found in endurance athletes. Currently, the debate about the superiority of a distinct TID model appears to focus on two models that were mostly found to be implemented by elite endurance athletes [4]: (i) The polarized TID consists of high volumes in Z1 and Z3, with only small proportions in Z2. Proportion of Z1 is generally higher than Z3 and proportion of Z3 is always higher than Z2. (ii) The pyramidal TID is characterized by high volumes of Z1 and gradually decreasing percentages in Z2 and Z3.

To date, research fails to give a clear answer, as to whether one of these TID models is superior. This non-uniformity appears to be partially founded in the different quantification methods that are implemented [6]. For example, a comparison of heart rate and velocity-based measures of TID in elite middle- and long-distance runners during an 8-week general preparation phase found a polarized TID when training was quantified by running speed (Z1: 79.9% ± 7.3%, Z2: 5.3% ± 4.9%, Z3: 14.7% ± 7.3%; Polarization-Index [Pol-Index]: 2.3 a.u.) and a pyramidal structured TID (Z1: 79.6% ± 7.2%, Z2: 17.0% ± 6.3%, Z3: 3.4% ± 2.0%; Pol-Index: 1.2 a.u.) when the training zones where quantified on HR [7].

Kenneally et al. recently created a novel way to determine endurance intensity zones in middle- and long-distance running based on the relative intensity of the athletes’ competition performance (percentage of race pace) [7, 8]. This new method is based on the fact, that previous research on the effects of different endurance training TID mainly analysed changes in physiological variables or time trial performances rather than actual competition performance, as the main aim of training. However, competition performance is influenced by multiple internal (e.g. central nervous system, biomechanics, cardiopulmonary system, etc.) and external factors (environment, tactics, etc.) and cannot be solely described by physiological variables [7]. In order to develop certain physiological systems (e.g. oxygen uptake, lactate threshold, exercise economy) it is well known that time spent in certain physiologically based intensity zones induce specific adaptation related to each zone [9]. Nevertheless, physiologically based intensity zones may be of limited value especially when training periods aim to specifically fine-tune the development of competition performance. Therefore, the race pace the race pace may serve as a better reference point to prescribe and analyse training intensity. The race pace-based approach aims to take different performance determinants of the competition performance into account [7], however this approach has its flaws as well. For example the TID of middle- and long-distance runners show markedly less interindividual differences when analysed based on race pace compared to TID analysis based on physiological benchmarks [7].

In contrast to endurance running, sprint kayaking imposes high demands on the upper-body endurance capacity of the athlete, as the athlete propels the boat-body-system against water resistance involving his/her relatively small upper-body muscles [10]. Differences in muscle mass, muscle fibre spectrum, oxygen extraction [11] as well as glucose and lipid oxidative capacity [11–13] between upper and lower body require different qualitative and quantitative training stimuli for distinct adaptation. From this perspective, the TID in kayak sprinting when compared to other leg-dominated sports should be different. Additionally, opposed to Olympic running-events with relatively high similarity of the courses, sports like kayak sprinting that are far more influenced by course characteristics (e.g. lake vs. artificial regatta course) and environmental factors (e.g. waves and water temperature) may not have the same potential to incorporate race pace-based velocity measures as a valuable tool for TID analysis [8].

So far, no study compared an internal and an external parameter-based method for determining TID over the course of a training cycle in kayak sprinting. Furthermore, no investigation implemented a race pace-based approach for TID analysis. Therefore, the current study aimed to retrospectively compare different TID quantification methods based on physiologically determined intensity zones using heart rate and velocity monitoring as well as race pace-based zone determination using velocity monitoring during an 8-week competition period in highly trained kayak sprint athletes.

## Materials and Methods

### Experimental Design

**Fig. 1:**
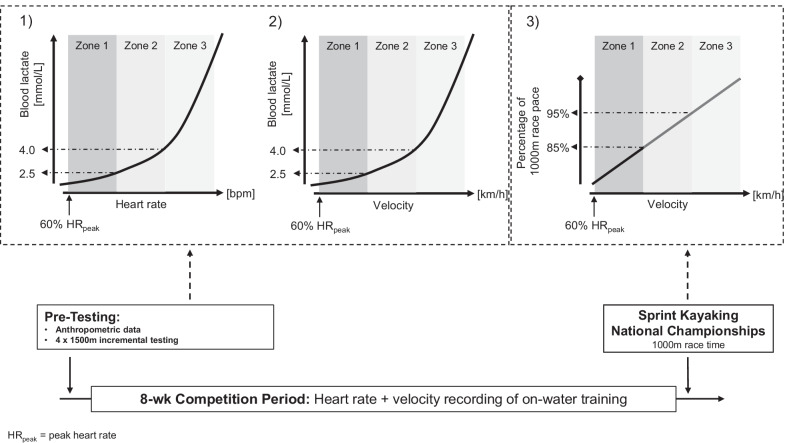
The overall study design with the three different methods of TID quantification. (1) Heart rate-based zones from blood lactate analysis during incremental testing. (2) Velocity-based zones from blood lactate analysis during incremental testing. (3) Velocity-based zones from 1000 m race time

This retrospective observational study was part of a 1-year study-project and took place during the season 2020. The present data were collected from June until August 2020. The observation comprised the final 8 weeks of the competition period before the German national championships which took place the week after data collection ended. Figure 1 illustrates the time course, methods involved and parameters obtained during the study period.

### Participants

The nine athletes were recruited from two different official performance headquarters of the German and/or North Rhine-Westphalian Canoe Federation. The key anthropometric, physiological and performance characteristics of the flatwater sprint kayakers are summarized in Table 1.

**Table 1:** Participants’ characteristics

P-ID	Sex	Age (years)	Height (cm)	Body Mass (kg)	Body Mass Index (kg/m^2^)	Peak oxygen uptake (ml/min)	Best times at Sprint Kayaking National Championships (s)		
							200 m	500 m	1000 m
1	Male	17	182	82.5	24.9	5275	< 38.3	< 105.1	< 224.1
2	Male	18	195	87.3	23.0	5347	< 40.1	< 105.6	< 225.6
3	Male	23	179	73.8	23.0	5209	n.d	< 105.8	< 222.2
4	Male	16	180	73.2	22.6	4137	n.d	< 107.1	< 230.4
5	Female	15	167	63.2	22.7	2714	n.d	< 124.4	< 257.2
6	Female	17	174	75.5	24.9	3068	< 47.2	< 123.6	< 264.9
7	Female	17	170	66.0	22.8	2931	< 42.9	< 119.3	< 261.7
8	Female	16	177	74.8	23.9	3330	< 44.2	< 121.7	< 257.9
9	Female	17	166	68.0	24.7	3038	< 46.8	< 124.2	< 265.9
Mean		17.3	177	73.8	23.6	3894	43.0	115.1	245.5
SD		2.3	9	7.7	1.0	1110	4.0	9.0	19.3

Four of the nine participants were members of the German Development Team and five were part of the Western German Regional Team. All participants competed on the highest national level in Germany. The athletes competed in kayaking for at least 6 years.

All were familiar with all testing procedures employed, having experienced frequent testing throughout their career. All procedures were approved by the institute’s ethics committee and conducted in accordance with the Declaration of Helsinki. After being informed in detail about the risks, benefits and procedures, all participants and their legal guardians gave their written consent to participate in this study.

### Determination of Intensity Zones

The HR (TID_Bla-HR_) and velocity (TID_Bla-V_) zones were based on the HR and velocity corresponding to certain blood lactate levels obtained from the incremental step test. The zones for TID_Bla-HR_ and TID_Bla-V_ were established based on the results of an incremental step test performed before the 8-week macrocycle. HR and velocity for each zone were based on previous categorization [5] with Z1 as HR and velocity corresponding to > 60% peak HR (HR_peak_) to HR at 2.5 mmol L^−1^ blood lactate; Z2 with HR and velocity corresponding to 2.5–4.0 mmol L^−1^ blood lactate and Z3 as an intensity with HR and velocity corresponding above 4.0 mmol L^−1^ blood lactate. The 2.5 and 4 mmol L^−1^ reference points refer to training zone prescriptions of the German Canoe Federation [14].

The TID based on race pace was also based on a three-zone model and based on previous categorization [7, 8] with Z1 as velocity corresponding from 60% HR_peak_ to 85% of race pace, Z2 as velocity corresponding from 86 to 95% of race pace and Z3 as velocity corresponding above 95% of race pace. These zone demarcations correspond to training recommendations by the Germany Canoe Federation for the three zones [14].

#### Polarization-Index

To quantify the individual level of polarization, we calculated a Polarization-Index (Pol-Index; a.u.) based on the time trained in each intensity zone. The Pol-Index was calculated as described in detail previously [15, 16] and calculated as follows:

1 $${\text{Polarization-Index}}\,\left( {{\text{a.u}}{.}} \right) = {\text{log}}_{{{1}0}} \left( {{\text{Z1}}/{\text{Z2}}*{\text{Z3}}*{1}00} \right).$$

A Pol-Index of > 2.0 a.u. is proposed to reflect a polarized TID.

#### Incremental Testing

All on-water testing took place on the regatta course in Duisburg-Wedau (Germany), the venue for several international canoe sprint championships, as well as the World Cup series of the International Canoe Federation (ICF). The participants were asked to refrain from all physical exercise for 12 h and exhausting exercise for 48 h prior to the experimental sessions, as well as to maintain their regular diet. In addition, all were instructed to avoid food intake 2 h prior to testing and were requested to arrive in a well hydrated state. Their diet and state of hydration were assessed with pre-test questionnaires.

The incremental test protocol involved 4 × 1500-m trials on-water at different intensities (i.e. 70%, 80% and 90% of HR_peak_, as well as an all-out effort) as described in detail previously [17]. Each incremental step was performed with a turn at 750 m to weaken the influence of wind and waves on performance. The time for each change in direction was extracted using the GPS-data. Previous investigations involving on-water incremental step testing in sprint kayakers showed acceptable to excellent reliability and validity of measurements of HR, oxygen uptake, blood lactate and stroke rate [18, 19] and strong correlations with performance [17].

The HR utilized for the first test was based on HR_peak_ obtained 6 to 8 weeks before with the same incremental on-water test protocol by the Western German Canoe Federation and each following test utilized the maximal heart rate of the previous testing. The 30–45 s interval between successive steps was the time required to sample capillary blood from the earlobe. All participants received continuous visual feedback from their HR monitor (Polar Wear Link System and V800 HR Monitor, Polar Electro OY, Kempele, Finland) mounted directly in front of them and averaged every second. The highest value during each incremental test was considered as HR_peak_. During each stage the stroke rate was self-selected.

Capillary blood was sampled from the right earlobe for analysis of lactate (Lactate Pro 2, Arkray KDK, Kyoto, Japan), at baseline and after each step. The HR and velocity associated with corresponding blood lactate concentrations were determined by linear interpolation between the two closest points, as in previous studies [20]. At the same time-points, rating of perceived exertion (RPE) was assessed employing the 6–20-point Borg scale [21].

### Heart Rate and Velocity Monitoring

The HR, velocity, distance and duration of every training session were collected from each sprint kayaker with a GPS-enabled watch (M430, Polar Electro Oy, Kempele, Finland) and the data were stored online (Polar Flow, Polar Electro Oy). To maximize measurement accuracy, HR was measured using a HR chest strap connected with the M430. All initiated the recording of each session with the beginning of the warm-up and stopped recording immediately with completion of cool-down. Afterwards, all datasets were visually checked for artefacts (e.g. flatline). Incomplete data files due to technical issues (e.g. low battery, HR or GPS-inaccuracy, etc.) and excluded from further analysis. Additionally, an experienced coach compared the number of recorded sessions with the online training diary of each athlete. From the data provided by the online version of the HR monitor (Polar Flow software), the total time spent in each HR and velocity zone for each training session was calculated.

#### Statistics

SPSS Statistics (Version 26; IBM Corp., Armonk, NY) was utilized for all statistical analysis. As performed earlier [6] a two-way analysis of variance with Bonferroni post hoc test was implemented to analyse for differences between TIDs derived from HR and velocity based on blood lactate-based zone demarcation as well as velocity based on race pace-based zone demarcation. Statistical significance was defined as *p* ≤ 0.05. Effect sizes were calculated as Cohen’s *d* values (Cohen, 1988), where 0.00–0.41 represents a small, 0.41–0.70 a moderate and ≥ 0.70 a large effect. In addition, 95% confidence intervals (95% CI) for Cohen’s *d* were calculated.

## Results

During the 8-week observation period, 557 single sessions with approximately 4500 km or 500 h of training were incorporated for analysis. The average training time per week within this period was 13.3 ± 1.4 h, with 7.9 ± 1.2 h of kayak-specific on-water training, 5.2 ± 0.7 h strength training, 1.8 ± 0.7 h other endurance training (e.g. running, swimming, cycling) and 1.1 ± 0.5 h of other activities (e.g. stretching, yoga).

**Fig. 2:**
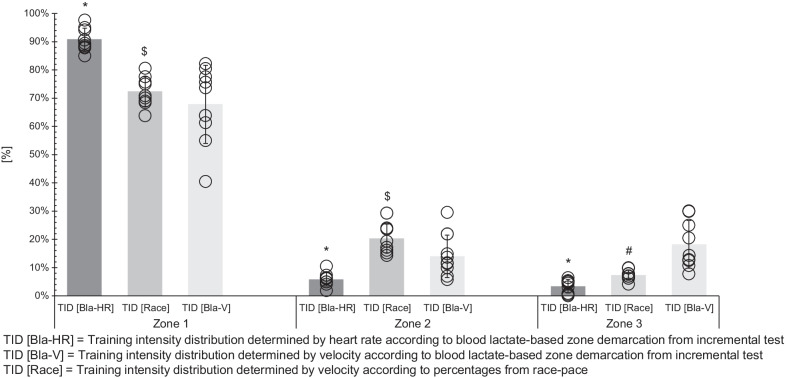
Mean (bars) and standard deviation (whiskers) of the different methods of TID for the three zones. The circles represent the percentage of total training time spent in each training zone for each athlete. Zones were determined by heart rate (TID [Bla-HR]) or velocity (TID [Bla-V]) according to blood lactate-based zone demarcation from incremental test and by velocity according to percentages from race pace (TID [Race]). * = sign. different from corresponding zone of TID [Bla-V] with *p* ≤ 0.01; $ = sign. different from corresponding zone of TID [Bla-HR] with *p* < 0.01; # = sign. different from corresponding zone of TID [Race] with *p* < 0.01

**Fig. 3:**
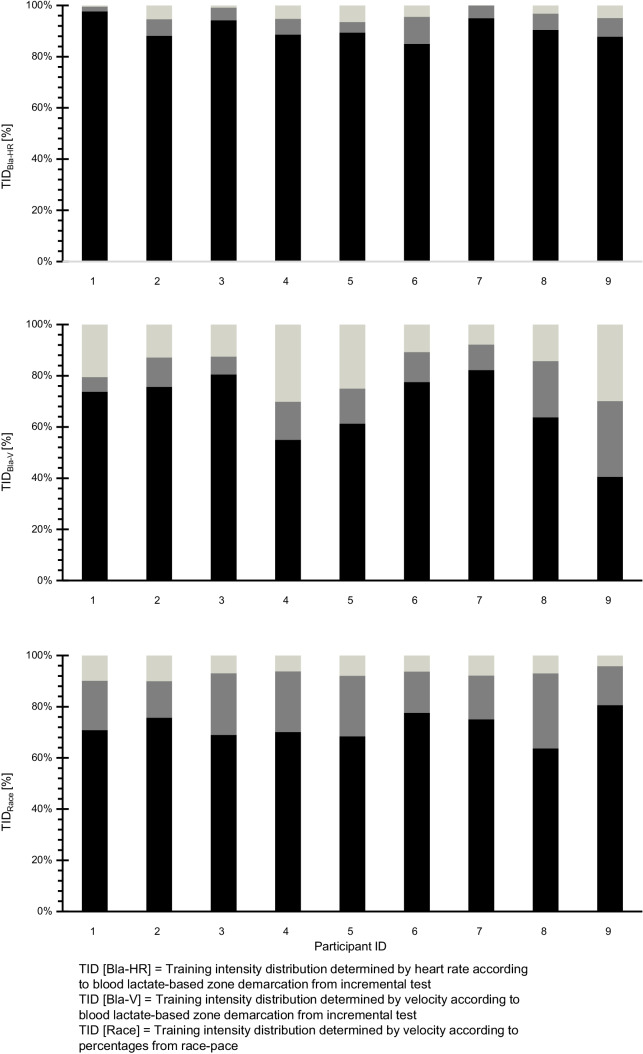
Training intensity distribution (percentage of total training time spent in each training zone) for each participant where zones were determined by heart rate (TID_Bla-HR_) or velocity (TID_Bla-V_) according to physiological testing and by velocity according to percentages from race pace (TID_Race_)

**Fig. 4:**
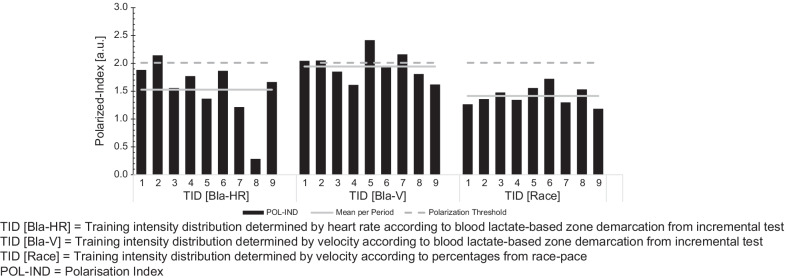
Individual Polarization-Index (POL-IND) for each analysed macrocycle and athlete. Mean = Group mean of Polarization-Index for the particular parameter. Polarization Threshold = Above a value of 2.0 in the Polarization-Index the TID is polarized (Treff et al., 2019)

The mean TID for the three different methods in each training zone during the observation period is summarized in Fig. 2. Figure 3 displays the TID for each participant. Additionally, Fig. 4 shows the Pol-Index for each participant and each TID determination method.

For TID_Race_ analysis, the mean velocity for the upper limit of Z1 and Z2 was 11.80 ± 0.94 km·h^−1^ and 13.27 ± 1.06 km·h^−1^, respectively. For the TID_Bla-HR_ and TID_Bla-V_ the upper limits were 12.37 ± 0.80 km h^−1^ (Z1) and 13.02 ± 0.82 km·h^−1^ (Z2). Mean HR for the upper limit of Z1 and Z2 for TID_Bla-HR_ was 173 ± 9 and 183 ± 10 beats per minute.

When employing TID_Bla-HR_ the mean plural of Z1, Z2 and Z3 were 91 ± 4%, 6 ± 2% and 3 ± 2% (Pol-Index: 1.6 ± 0.4). For TID_Bla-V_ the fractions were 68 ± 14%, 14 ± 8% and 18 ± 8% (Pol-Index: 1.9 ± 0.3), with each fraction differing to TID_Bla-HR_ (Z1: *p* < 0.01, *d* = − 2.23, 95% CI − 3.28 to − 0.97; Z2: *p* = 0.01, *d* = 1.37, 95% CI 0.29 to 2.32; Z3: *p* < 0.01, *d* = 2.57, 95% CI 1.23 to 3.67). The Z1, Z2 and Z3 plural of TID_Race_ were 73 ± 5%, 20 ± 5% and 7 ± 2% (Pol-Index: 1.4 ± 0.2), respectively. Z1 and Z2, but not Z3 of TID_Race_ zone fractions differed when compared to TID_Bla-HR_ (Z1: *p* < 0.01, *d* = 4.20, 95% CI 2.40 to 5.60; Z2: *p* < 0.01, *d* = − 3.68, 95% CI − 4.97 to − 2.03) and compared to TID_Bla-V_ only Z3 was different (*p* < 0.01, *d* = 1.89, 95% CI 0.70 to 2.89).

On an individual level the ranges in each zone and with each TID determination method were as follows: For TID_Bla-HR_ Z1 to Z3 ranged from 85 to 98%, 2 to 11% and 0.1 to 6% (Pol-Index: 0.3–2.1), for TID_Bla-V_ 41 to 82%, 6 to 30%, 8 to 30% (Pol-Index: 1.6–2.4) and for TID_Race_ 64 to 81%, 14 to 29% and 4–10% (Pol-Index: 1.2–1.7), respectively. With TID_Bla-HR_ all but one of the athletes revealed a pyramidal TID (Pol-Index < 2.0) with extremely high fractions in Z1 (> 85%), while only one athlete (P-ID 2) highlighted a polarized TID with a Pol-Index of 2.1 and only 2% difference between Z2 (4%) and Z3 (6%). With TID_Bla-V_ three athletes (P-ID 2, 5, 7) showed a polarized intensity distribution (Pol-Index ≥ 2.1) and two athletes revealed a polarized like TID pattern, i.e. higher proportions of Z3 than Z2, with one of the two (P-ID 6; Pol-Index = 1.9) revealing almost identical fractions for Z2 (11%) and Z3 (13%) and the other (P-ID 4; Pol-Index = 1.6) relatively low proportion in Z1 (55%). Additionally, one athlete (P-ID 9; Pol-Index: 1.6) with TID_Bla-V_ had an almost uniform distribution of the three zones with 40, 30 and 30% in Z1-Z3. TID_Race_ analysis shows a pyramidal distribution for each athlete.

## Discussion

The current study aimed to retrospectively compare different TID quantification methods based on physiologically determined intensity zones using HR and velocity monitoring as well as race pace-based zone determination using velocity monitoring during an 8-week competition period in highly trained kayak sprint athletes. This retrospective analysis is to best of our knowledge the first observation in the literature comparing these different quantification methods for TID in kayak sprinting.

The major findings were:

(i) TID_Bla-HR_ significantly differed in each intensity zone from TID_Bla-V_ and in Z1 and Z2 from TID_Race_.
(ii) TID_Bla-V_ differed in Z3 fractions when compared TID_Race_.
(iii) Depending on the quantification method and on group level TID_Bla-HR_ and TID_Race_ yielded rather pyramidal TID whereas TID_Bla-V_ resulted in more polarized TID.
(iv) On an individual level TID_Race_ was pyramidal for each athlete, TID_Bla-HR_ was pyramidal for eight athletes and polarized for one athlete and TID_Bla-V_ revealed to be polarized for three athletes, polarized like for two athletes, uniform for one and pyramidal for three athletes.

### Intensity Distribution Based on TID_Bla-HR_ and TID_Bla-V_

The fraction of each zone with TID_Bla-HR_ and TID_Bla-V_ differed substantially. TID_Bla-HR_ revealing a pyramidal TID for Z1, Z2 and Z3 (91 ± 4%, 6 ± 2%, 3 ± 2%; Pol-Index: 1.6 ± 0.4), whereas TID_Bla-V_ showed a more polarized TID (68 ± 14%, 14 ± 8% and 18 ± 8%; Pol-Index: 1.9 ± 0.3). Research concerning both TID quantifications methods is scarce, and so far, the only existing studies were implemented in running [6] and cycling [22], e.g. endurance sports that mainly implement the lower body for propulsion. In contrast, sprint kayaking imposes high demands on the endurance capacity of the athletes’ upper body, as the athlete propels the boat-body-system against water resistance involving relatively small upper-body muscles [10]. Differences in muscle mass, muscle fibre spectrum, oxygen extraction [11] as well as glucose and lipid oxidative capacity [11–13] between upper and lower body require different qualitative and quantitative training stimuli for distinct adaptation. Recent studies in elite cross-country skiers investigated upper and lower-body muscles and found upper-body muscles to be less capable to oxidize fat and to rely more on carbohydrate oxidation than lower-body muscles [11, 12]. Thus, it may seem plausible to assume that kayakers may not be able to perform as much volume in the higher intensity zones (Z2, Z3), as arm-glucose storages depleted earlier and therefore kayakers (have to) implement more time in Z1 compared to whole-body and/or lower-body sports.

Despite the differences in muscles involved in propulsion, data from endurance running [6] and endurance cycling [22] are partly in agreement with the current findings. Bellinger et al. analysed the TID based on HR and running speed corresponding to below first ventilatory threshold, between first and second ventilatory threshold and above second ventilatory threshold of fourteen highly trained middle-distance runners during an 8-wk training period [6]. In the latter investigation, TID differed especially in time spent in Z2 (HR: 17.0 ± 6.3% vs. speed: 5.3 ± 4.9%) and Z3 (HR: 3.4 ± 2.0% vs. speed: 14.7 ± 7.3%), resulting in a polarized TID for the velocity-based and a pyramidal TID for HR-based zone quantification. In line with the results of Bellinger and colleagues [6], another study [22] found higher fractions of Z3 for power output-based TID (11.5 ± 2.5%) quantification compared to HR-based TID quantification (4.4 ± 2.0%), when comparing a three-zone TID model based on aerobic and anaerobic threshold performance in road cyclists during a 10-week training period.

The differences in Z2 and Z3 between HR- and velocity/power-derived TID quantification shown in the previous studies [6, 22] and our findings between TID_Bla-HR_ and TID_Bla-V_ are most likely attributable to the delayed HR-kinetics at the beginning of an (intense) exercise bout. Especially, with short high-intensity bouts (< 30 s) the HR response is inertial and does not reflect the entire 30-s effort [5, 23]. Thus, lower fractions of Z3 and higher fractions of Z2 with TID_Bla-HR_ are possible. As a consequence, TID analysis based on HR is likely impractical to reflect the neuromuscular demand of high-intensity short-duration efforts, while external measures such as velocity seem to reflect these demands more accurately [22, 23]. In fact, in training regimes of sprint kayaking short intervals with maximum intensity are frequently implemented as the (i) start phase performance has an important impact on race performance [10] and (ii) work performed during 30 s all-out kayaking is correlated with performance over all three Olympic distances [24] as reflected in the higher fractions of Z3 for velocity-based measures in the current study. The underestimation of Z3 by HR-monitoring is further confirmed by two recent studies [25, 26] investigating kayak sprinters and comparing post-session intensity analysis based on HR and power output. In this case, HR-based training monitoring also underestimated high-intensity training loads and overestimated training time in Z2. Accordingly, in phases with focus on lower intensities and longer distances covered in each exercise bout (e.g. general preparatory period) TID would differ less between HR and external measures compared to phases with more short high-intensity training sessions (e.g. competition and taper periods) [22].

In the present analysis quantification of Z1 markedly differed to the findings of previous studies [6, 22]. The current study shows significantly higher proportions of Z1 TID_Bla-HR_ compared to TID_Bla-V_, while Bellinger et al. [6] and Sanders et al. [22] found no difference for Z1 fractions between HR and velocity monitoring. The following explanation may account for the inconsistent data: kayakers often perform their long-distance endurance sessions in groups and usually one kayaker leads, and the training partners follow situated to the left and right side behind the leader and/or directly behind him/her [27]. This arrangement is called “wash-riding” as the kayakers behind the leader are assisted in their forward paddling by the force of the “wash” (i.e. undertow) generated by the boat of the leading kayaker. Previous analysis found that wash-riding saves up to 31.9% of energy, which is also associated with markedly decreased blood lactate and heart rate values during a constant endurance session [27]. As it is common for kayakers to change the group leader each kilometre or each 5 min, the position alterations allow the group to maintain a relatively high velocity over an entire session reflecting a Z2-velocity while keeping the HR low (i.e. Z1). Therefore, from a practical point of view, it is likely that Z1 contribution is underrated and Z2 contribution overrated when employing TID_Bla-V_. Future studies on TID in kayaking should consider carefully, if the athletes should be allowed to perform wash-riding.

Consequently, when analysing training intensity in kayak sprinters, both quantification methods TID_Bla-HR_ and TID_Bla-V_ may have crucial downsides for TID analysis and further decision making. Based on the present data, a combination of both methods may yield value for TID quantification in kayak sprinting with TID_Bla-HR_ implemented to determine Z1 and Z2 and TID_Bla-V_ for Z3. Further research is warranted in this regard.

In summary, previous retrospective TID_Bla-HR_ analysis seems to yield more pyramidal TID [3, 6, 28–33] whereas when integrating external measurement (e.g. velocity or power output) [6] or the session goal method [3, 34] with complemented HR measurement the TID reveals to be more polarized. This pattern of TID-dependence on the implemented quantification method applies over a range of different sports like running [6, 28], cycling [29, 31, 33], cross-country skiing [3], rowing [30].

### Intensity Distribution Based on TID_Race_

The analysis of TID_Race_ showed a pyramidal TID pattern on the group level. Most interestingly, this pattern was found consistently for each athlete (Fig. 3C). This high congruency among the group in TID was not evident with the other two quantification methods, i.e. TID_Bla-HR_ and TID_Bla-V_ (Fig. 3A, B). Similarly, Kenneally et al. [7] concluded that TID is subject to less interindividual variation among middle- and long-distance runners when employing race pace compared to the physiological benchmark-based TID quantification, probably because training during the competitive period targets mainly to develop race pace. In a review article, the same research group [8] analysed TID of different middle- and long-distance runners employing either HR- or velocity-based TID and calculated training intensity zones relative to the targeted race pace and found race pace to be a more important factor in the design of training programmes than physiological zone demarcation and founded this in the high similarity between athletes TID when based on race pace.

However, it remains questionable if the consistency in TID_Race_ zone proportion among athletes favours argument for race pace-based zone quantification, as, e.g. so far, no study analysed coherences between race pace-based zone quantification and performance-related measures. Additionally, the TID_Race_ in our analysis resulted in a markedly wider speed range for Z2 (1.47 km/h ± 0.12 km/h) when compared with the physiologically based TID_Bla-HR_ and TID_Bla-V_ (0.66 km/h ± 0.27 km/h), which is in accordance with previous research [7, 35] and most probably explains the higher homogeneity of TID_Race_. The markedly wider speed range for Z2 in TID_Race_ explains the shift from higher fractions in Z3 and lower fractions in Z2 in the physiological approach to comparably higher fractions in Z2 and lower fractions in Z3 compared to the race pace approach, which was similarly found in middle- and long-distance runners [7]. The wide range of Z2 in the race pace approach certainly includes intensities that exceed the “threshold” zone and thus, possibly provides a wider range of training stimuli [7]. If coaches are not aware of the higher intensities included in Z2 when using the current approach of race pace-based training intensity prescription, the use of the method potentially may lead to an unintended overload. Although the TID_Race_ assists coaches and athletes with feedback about the training intensity relative to competition performance (and therefore representing a marker with high specificity especially in the final weeks prior to competitions), the TID analyses based on physiological zone demarcation is important to receive knowledge about the development of the different energy systems during the training process.

Furthermore, especially for the sport of sprint kayaking, TID_Race_ reveals several difficulties: (i) as an outdoor sport, kayaking race performance is strongly affected by weather conditions (e.g. water temperature, wind, waves, etc.) and type of waters (e.g. artificial regatta course, lake, river, etc.) where training and competition are performed, leading to substantial variation in performance times. This variation subjects a session-to-session as well as competition-to-competition comparison of speed sensitive to errors. Future research should provide recommendations for kayak-specific training intensity prescription/analysis which consider these variations. (ii) In kayaking it is quite common to train for and compete over, at least, two of the three Olympic distances (200 m, 500 m, 1000 m). This variation can lead to differing intensity prescriptions using race pace, which most probably is no issue within Z3 sessions, where the session aim is mostly specific to the race distance. However, during Z1 and Z2 sessions, the session aim must not be clearly assigned to a specific race distance. As, in the current study, race pace-based TID was solely based on 1000 m race pace, Z3 fractions would have been even lower in favour of Z2 if each sessions intensity would have been calculated on the race distance targeted in the individual session. For example, an athlete with a race pace of 15.00 km/h (= 4:00 min/km) over the 1000-m distance and of 16.36 km/h (= 3:40 min/km) over the 500-m distance, who trains 2-min-intervals at a pace of 14.50 km/h (= 4:08 min/km), would have a Z3 session when analysing based on 1000-m race pace (14.50 km/h = 97% of 1000-m race pace), but only a Z2 session when 500-m race pace (14.50 km/h = 89% of 500 m race pace) is the benchmark for analysis. A possible compromise could be to use the race pace of the main competition which an athlete is primarily focusing on. If an athlete competes in two different disciplines (e.g. 200 m and 500 m), one might consider using the race pace of an intermediate distance (e.g. 350 m) to provide a TID quantification. A further method could be the training intensity prescription and/or analysis based on the individual session aim (e.g. the individual race pace of the targeted race distance in each session). This approach has been discussed previously [23] and focusses on the training of the athletes’ entire locomotor profile (e.g. start phase performance and anaerobic speed) instead of solely focusing on developing a single physiological performance parameter (e.g. speed at anaerobic threshold). As competition performance is determined by the interaction of different performance-related parameters [24], this method, oriented on the race distance targeted in the individual session, may provide a more goal-orientated approach compared to training prescription solely based on physiological parameters. However, this method is suggested to be mostly valuable for experienced athletes and coaches who know the best performance times on several distances and have low intersession performance variation [23]. Ultimately, in contrast to TID_Bla-HR_ and TID_Bla-V_, the aforementioned examples for adjusting the quantification of TID_Race_ demonstrate the flexibility of this method for adapting specific aims and circumstances of each athlete in his/her preparation.

Since TID_Bla-HR_, TID_Bla-V_ and TID_Race_ demonstrate up- and downsides it appears important for researchers and coaches to raise the question about “what is the main aim of the current training phase?”. The race pace-based approach might be of value during competition periods, but during periods with less emphasis on specific race pace development the threshold-based approach might be more valuable. Additionally, a shift from a TID_HR_ and TID_Bla_ during preparatory periods to a race pace-based approach during the competition period would be in accordance with one of the most accepted principles of training, which is the principle of specific adaptations to the imposed demands [36] (SAID-principle). The SAID-principle considers the versatility of influences, beside the traditional metabolic considerations, that impact race performance. Especially, as until today, research failed to find the one optimal TID model and/or physiological parameter to predict performance [3, 8], race pace-based intensity prescription may provide a better stimulus to concurrently develop key endurance variables in the final preparation for competition performance [8].

## Limitations

Certain limitations associated with the current investigation warrant consideration. The relatively small number of subjects may have affected the power of the statistical analysis. However, it is difficult to conduct investigations of this nature on large numbers of highly trained athletes who must prioritize their own individual training and competition. We believe that the current investigation provides useful information for coaches and researchers in this specific field. Moreover, it is important to note that on-water TID analysis does not provide a comprehensive evaluation of the stress an athlete is exposed to. Non-specific training (e.g. resistance training, dry-land endurance training, etc.) and various additional factors (e.g. sleep, daily physical activity, nutrition, etc.) have an impact on overall training stress and need to be considered when holistically evaluating an athletes training stress [37, 38]. Furthermore, as discussed earlier, when analysing data from an outdoor sport like kayaking, one must be aware of the unstable nature of the sport that is constantly influenced by water and weather conditions. Thus, a future challenge for research is to provide models to minimize the impact of environmental fluctuations on TID analysis.

## Conclusion

The current study aimed to retrospectively compare TID quantification based on physiologically determined intensity zones as well as race pace-based zone determination using velocity monitoring during the competition period in highly trained kayak sprint athletes. Based on the present data and observation period we conclude that the method of quantification affects the calculation of the TID in highly trained sprint kayakers. The most noticeable difference between the TID determination method is the comparably low interindividual variation in TID for TID_Race_. Depending on the aim of the analysis and training goal TID_Race_, TID_Bla-HR_ and TID_Bla-V_ have advantages as well as drawbacks and may be implemented in conjunction to maximize adaptation.

## Data Availability

As the athletes in this study are still competing on national and international level, supporting data are not available.

## Abbreviations

a.u.: Arbitrary unit
B_la_: Blood lactate
CI: Confidence interval
GPS: Global position system
P-ID: Participant identification
Pol-Index: Polarization-Index
RPE: Rating of perceived exertion
TID: Training intensity distribution
TID_Bla-HR_: Training intensity distribution derived from heart rate based on blood lactate concentrations of incremental step testing
TID_Bla-V_: Training intensity distribution derived from velocity based on blood lactate concentrations of incremental step testing
TID_Race_: Training intensity distribution derived from velocity based on 1000-m race pace
HR: Heart rate
HR_peak_: Peak heart rate
VO_2_: Oxygen uptake
VO_2max_: Maximum oxygen uptake
Z: Zone
